# Knowledge and Attitude towards Pain Management among Nurses Working at University of Gondar Comprehensive Specialized Hospital, Northwest Ethiopia

**DOI:** 10.1155/2020/6036575

**Published:** 2020-03-18

**Authors:** Bikis Liyew, Ambaye Dejen Tilahun, Netsanet Habtie Bayu, Tilahun kassew

**Affiliations:** ^1^School of Nursing, College of Medicine and Health Sciences, Department of Emergency and Critical Care Nursing, University of Gondar, Gondar, Ethiopia; ^2^School of Nursing, College of Medicine and Health Sciences, Department of Comprehensive Nursing, University of Gondar, Gondar, Ethiopia; ^3^School of Medicine, College of Medicine and Health Sciences, Department of Psychiatry, University of Gondar, Gondar, Ethiopia

## Abstract

*Introduction*. Pain is unpleasant sensory and emotional experience associated with actual and potential tissue damage. Inadequate pain management has been shown to affect patient outcomes by potentially increasing hospital stay and delaying recovery; thus, the management of pain has major implications for nursing. The study was aimed to identify gaps in knowledge and attitude of nurse's level of pain management.

**Objective:**

This study aimed to assess knowledge and attitude towards pain management among nurses working at University of Gondar comprehensive specialized referral hospital, Northwest Ethiopia, 2019.

**Methods:**

Institution-based cross-sectional study was carried out during May 20–30, 2019. A stratified random sampling technique was used to select 422 nurses. Hospital departments were classified into 5 main strata having nearly the same working conditions: (1) Internal Medicine, (2) Pediatrics, (3) Surgical, (4) Outpatient Clinics, and (5) Emergency and Intensive Care Departments. The proportional allocation was taken from each stratum, and then a simple random technique was applied. Descriptive statistics like frequency, mean, median, standard deviation, and percentage were used to describe the distribution of data. Independent samples *t*-test was used in comparing the gender knowledge and attitude mean score of the nurses towards pain management. One-way ANOVA was also used in determining the differences between knowledge and attitude towards pain management with sociodemographic characteristics at the *p*=0.05 level of significance.

**Result:**

A total of 411 study participants were involved in this study. More than half of the nurses, 225 (58.1%), were males; 308 (79.5%) of them were in the age category of 19–29 years. The magnitude of good knowledge towards pain management among nurses was 66.9% with a mean score of 7.14 (1.74 SD). The magnitude of favorable attitude towards pain management among nurses was 51.7% with a mean score of 49.33 (7.13 SD). *Conclusion and Recommendation*. This study revealed that nurses working at University of Gondar hospital had good knowledge and a lower level of attitude towards pain management than those reported in previous studies. Thus, the situation demands various educational and quality improvement initiatives that could enhance the nurse‘s knowledge and attitude in the area of pain management.

## 1. Introduction

Pain is an unpleasant sensory and emotional experience associated with actual and potential tissue damage [1]. Pain is a stressful experience that is considered to be a global health problem. Studies have reported that 55% to 78.6% of inpatients experience moderate-to-severe pain. There are still problems regarding pain management despite countless training courses, application strategies, and multidisciplinary pain teams [2]. Nurses are often the only ones who may hear of pain endured by the patients and who carry out the advice of the physicians on pain management. Therefore, their knowledge and attitude are very important in pain management. The gap in knowledge about pain assessment and management, inability to assess pain, and poor communication between the patient and the healthcare provider lead to ineffective pain management and approximately 79% of the hospitalized patients suffer from it [3].

An adequate level of knowledge and a positive attitude are essential components in the delivery of pain management [4]. A descriptive cross-sectional study in Saudi Arabia shows that there were lack of knowledge and poor attitudes among nurses towards pain management when dealing with ICU patients. Pain is the major symptom that brings patients to the healthcare setting and is also the commonest symptom that hospitalized patients encounter in general and in surgical settings in particular [5]. Pain is a global health issue that requires the attention of the health community [6]. Another study showed that 79% of inpatients suffer from pain. Nurses as the healthcare professionals are often closer than others to the patients in all services including in surgical settings; therefore, they are highly responsible for effective pain management and must advocate for the patients [7].

Inadequate assessment and treatment of pain continue to be an issue in the care provided in the healthcare system in Ethiopia. A nurse is a key person who can improve the quality of pain management and who can provide nursing care to sufficiently meet the patient's needs [8]. Nurses with stronger knowledge and attitude lead to better pain management, improved outcomes, and higher patient satisfaction scores [9]. In clinical settings, nurses play a vital role in pain assessment and management and must be knowledgeable regarding how to best assess and manage the pain [10].

However, inadequate pain management is evident across all ages; previous reports have found that children receive less analgesia than adults in comparable situations, where significant numbers of hospitalized children experience unacceptable levels of pain resulting from lack of knowledge with pain management practice among healthcare providers and the myths that infants and children do not feel pain (suffer less from it than adults) [11].

Inadequate pain management has been shown to affect patient outcomes by potentially increasing hospital stay and delaying recovery; thus, the management of pain has major implications for nursing [11]. This can affect patients' physiological and health safety. This study shortens the gap of information about knowledge and attitude of pain management among nurses in the area. Therefore, assessing the knowledge and attitude of nurses is important to improve the pain management process; there by reducing morbidity and mortality associated with pain would be possible. Hence, this study aims to assess knowledge and attitude towards pain management among nurses working at University of Gondar specialized hospital, Northwest Ethiopia.

## 2. Main Text

### 2.1. Methods

#### 2.1.1. Study Design, Area, and Period

An institution-based cross-sectional study was conducted from May 20 to May 30, 2019, at Gondar University comprehensive specialized referral hospital. This hospital is in Amhara regional state, Northwest Ethiopia. The source populations for this study were all nurses working in Gondar University comprehensive specialized Hospital. The study populations in this study were all nurses who are working at Gondar University comprehensive specialized Hospital during the period of data collection. Nurses who had work experience below six months or were on annual break or maternal leave and administrative staffs were excluded from the study.

#### 2.1.2. Sample Size Determination and Procedure

The required sample size was determined by using a single population proportion formula by considering 95% CI, 5% margin of error, Proportion = knowledge = 58% [12], and 10% of the calculated sample size was added to compensate nonresponse rate. Finally, a total of 411 nurses were recruited in the study. A stratified random sampling technique was used to select 422 nurses. Hospital departments were classified into 5 main strata having nearly the same working conditions: (1) Internal Medicine, (2) Pediatrics, (3) Surgical, (4) Outpatient Clinics, and (5) Emergency and Intensive Care Departments. The proportional allocation was taken from each stratum and then a simple random technique was applied.

#### 2.1.3. Operational Definition

*(1) Knowledge*. It means the nurses' understanding of pain management based on their experience.

*(2) Good Knowledge*. It is the knowledge status of nurses when they scored mean and above.

*(3) Poor Knowledge*. It is the knowledge status of nurses when they scored less than the mean.

*(4) Attitude*. It refers to the nurses' behavior and way of acting towards effective pain management.

*(5) Favorable Attitude*. It is the category of nurses when they scored mean and above value.

*(6) Unfavorable Attitude*. It is the category of nurses when they scored less than the mean value.

*(7) Pain Management*. The nursing practice of assessing, diagnosing, planning, intervening in, and evaluating patient's pain in the hospitals.

#### 2.1.4. Data Collection Tools and Techniques

Data were collected using a structured and pretested self-administered questionnaire. The questionnaire was adopted and modified from previous studies [13,14]. Initially the 16-item questionnaire was developed by Lebovits et al. which has not been yet analyzed psychometrically [15]. 21 items of nurses' knowledge and attitude survey (NKAS) were psychometrically tested by Visentin et al [13] and 4 items were added by the authors to determine the nurses' knowledge and attitude towards pain by three nursing experts with in-depth discussion. The questionnaire consisted of three sections. The first section collected sociodemographic data. The second section focused on nurses' knowledge towards pain management, and the third section focused on the nurse's attitude towards pain management. The questionnaire was comprised of 25 items in English, including 12 knowledge questions, and the interviewees were asked to reply in two ways: 1 = yes; 0 = no, with a total range of “0–12”; and they were asked to answer 13 attitude questions according to five-point Likert scales: from “strongly agree = 5” to “strongly disagree = 1” with total score range of “13–65”.

Answers were evaluated considering the extent to which they were compatible with pain therapy standards commonly acknowledged by the international pain management guidelines. The tool was prepared and administered in English. The internal consistencies of the 25 knowledge and attitude assessment items were very good as evidenced by the overall Cronbach's Alpha and reliability of the items was *α* = 0.87. Data were collected by BSc nurses and the investigator was responsible for the overall management of the study, the development of the final questionnaire, and identifying and assignment of data collectors and supervisors in the group.

#### 2.1.5. Data Quality Control

Instruments for this study were developed by modifying standard questioners with great care and a pretest was conducted using 5% [16] of the population on nurses who were working at Debark Hospital. Appropriate modifications were made after analyzing the pretest result before the actual data collection. The training was given for data collectors for one day to ensure all the group members had the same information about the study instrument and follow the same survey administration procedures. The principal investigators collected the filled questionnaire and checked for missed values and completeness daily.

#### 2.1.6. Data Processing and Analysis

The collected data were entered into Epi Info version 7.2.2 and analyzed by using SPSS Version 23. The data were cleaned for inconsistencies and missing values and modifications were made as needed. Descriptive statistics were carried out. Simple frequencies were used to see the overall distribution of the study subject with the variables under study. Finally, the results were presented in texts, graphs, and tables. Independent samples *t*-test was used in comparing the gender knowledge and attitude mean score of the nurses towards pain management. One-way ANOVA was also used in determining the differences between knowledge and attitude towards pain management with sociodemographic characteristics at the *p*=0.05 level of significance.

## 3. Result

### 3.1. Sociodemographic Characteristics of the Study Participants

In this study, 387 participants were included in the analysis with a response rate of 94.2%. 225 (58.1%) of the participant nurses were males; nearly two-thirds, 243 (62.8%), of the participants were single and 324 (83%) were BSc holders. The mean ages of the respondents were 27.25 (±5.22 SD) with minimum and maximum ages of 19 and 61 years, respectively. Respondents had a mean of 3.28 years (±2.35 SD) of work experience with minimum and maximum of 1 and 27 years, respectively (Table 1).

### 3.2. Knowledge of Nurses towards Pain Management

Nearly two-thirds, 255 (65.9%), of the respondents answered that narcotics can cause respiratory depression and that they should not be used in pediatric patients. 269 (69.5%) study participants answered incorrectly as it may not often be useful to give a placebo to a patient in pain to assess if he is genuinely in pain. 227 (58.7%) study participants responded that patients having severe chronic pain often need higher dosages of pain meds than patients with acute pain. More than half, 227 (58.7%), of study participants were answered that the preferred route of administration of narcotic pain relievers to patients with pain is not intramuscular (see Table 2).

**Figure 1:**
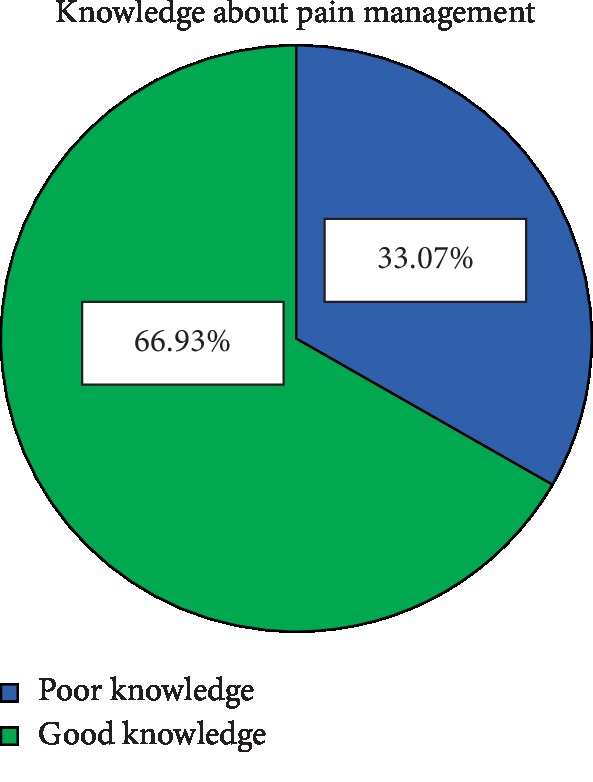
The overall knowledge of nurses about pain management at University of Gondar comprehensive specialized referral hospital, Northwest Ethiopia, 2019.

Overall, 259 (66.9% with 95% CI (62.5%, 71.6%)) of respondents had good knowledge about pain management with a mean of 7.14 (±1.74 SD). The maximum and the minimum knowledge score that was correctly answered was 12 and 1, respectively (see Figure 1).

Using independent sample *t*-test, there was no significant difference between males (mean = 7.16) and females (mean = 7.11) in the mean score of nurses' knowledge towards pain management with *p* value = 0.765. In one-way ANOVA analysis, there was no significant difference between marital status (*p*=0.868), qualification (*p*=0.901), unit/ward (*p*=0.364), work experience (0.711), and age (*p*=0.236) as regards the mean score of nurses' knowledge towards pain management.

### 3.3. The Attitude of Nurses towards Pain Management

144 (37.2%) of the respondents agreed that a lack of pain expression does not mean a lack of pain. Out of the total of respondents, 164 (42.4%) of study participants agreed that when a patient requests increasing amounts of analgesics to control pain, this usually indicates that the patient is psychologically dependent. From the respondents, 152 (39.3%) of the study participants agreed that the most accurate judgement of the intensity of the patient's pain is the patient himself/herself. 175 (45.2%) of respondents agreed that, for effective pain treatment of cancer pain, it is necessary to continuously assess the pain and the efficacy of therapy (see Table 3).

**Figure 2:**
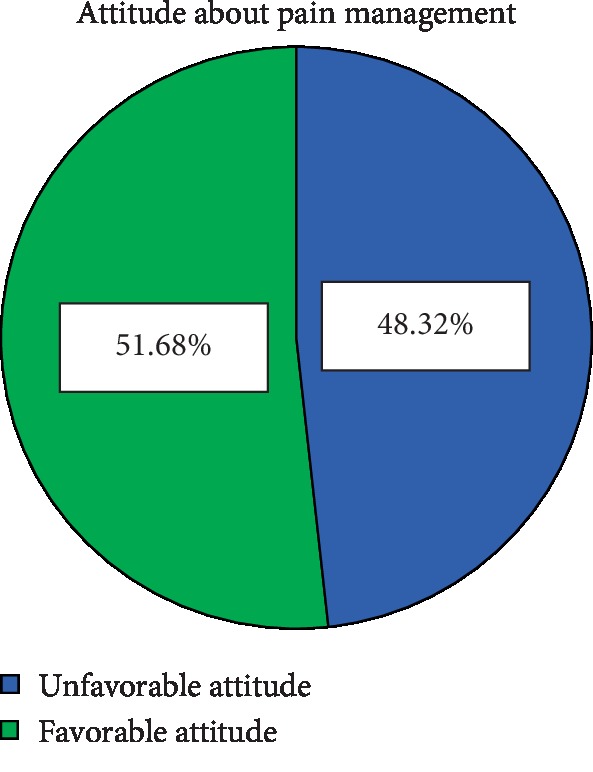
Attitude of nurses towards pain management, UOGCSH, Northwest Ethiopia, 2019.

The overall result of this study showed that 200 (51.7% with 95% CI (46.3%, 56.1%)) of the respondents had a favorable attitude towards pain management with a mean score of 49.33 (±7.13 SD). The minimum and maximum attitude scores of the study participants were 21 and 65, respectively (see Figure 2).

In an independent sample *t*-test, there was a significant difference between males (mean = 50.06) and females (mean = 48.31) in the mean score of nurses' knowledge towards pain management with *p* value = 0.02. In one-way ANOVA analysis, there was no significant difference between marital status (*p*=0.99), qualification (*p*=0.82), unit/ward (*p*=0.20), work experience (0.10), and age (*p*=0.66) as regards the mean score of nurses' attitude towards pain management.

## 4. Discussion

### 4.1. Knowledge of the Study Participants about Pain Management

In this study, 66.9% (95% CI (62.5%, 71.6%)) of nurses had good knowledge about pain management. This finding was consistent with the studies done in Tasmania (Island States of Australia) where 71% [17] of their study participants had good knowledge of pain management. The possible reason for the similarity between the current study and Tasmania's might be using a similar study design (cross-sectional) and study population (nurses).

The result of this study was lower than the studies done in Uganda, 75% [18], Saudi Arabia 87.5% [19], United Kingdom (UK) 73.8% [20], and Chicago, United States of America, 74% [21], where these percentages of the study participants had good knowledge towards pain management. The possible justification for this difference might be due to socioeconomic status, study setting, sample size, and use of different data collection tools. In the current study, Ethiopia is regarded as a low-income country compared to other listed countries. This indirectly affects the quality of healthcare education, so that is why we got a lower magnitude of good knowledge. When comparing the sample size and data collection tool, our study has larger sample size (*n* = 387) and used tool was a questionnaire of 12 knowledge and 13 attitude items; whereas in the study done in Uganda the sample size was lower (*n* = 67) and the used tool was a questionnaire of 16 knowledge and 12 attitude items, which resulted in the magnitude difference. In a study held in Saudi Arabia, the setting was multicenter (3 hospitals) and the clinical setting of participants was surgical and medical ward, whereas our study was in a single institution and included all clinical setting areas. A study done in the United Kingdom had a comparative cross-sectional study design but our study design was descriptive cross-sectional. The USA study used a mail data collection technique by using 52 items for the assessment of knowledge among 1000 emergency nurses, whereas our study used self-administered, 25-item knowledge and attitude questionnaire among 387 nurses working in different clinical areas. Those reasons might have resulted in a lower magnitude of good knowledge in this study.

The result of this study also showed that nurses' knowledge towards pain management was higher than that in the studies done in Mekelle, Ethiopia, 58.6% [22], Zimbabwe, 35.5% [16], Iran, 46.6% [23], Turkey, 38.2% [12] and 39.5% [24], Australia, 24% [25] and Malaysia, 25% [4]. The possible justification of this finding which is higher than the above studies might be due to differences in study setting, the difference in sample size, different instruments used, and the difference in the outcome rating they used; for example, in Malaysian study, the knowledge was rated as high, moderate, and low. These may change the figurative interpretation of this study as good and poor knowledge. The difference might also be due to the fact that, unlike study in Mekelle, Ethiopia (only Bachelor and diploma), nurses with a master degree were involved in this study. These may relatively increase the knowledge status of the study participants. Moreover, the sample size variation among the two studies could bring the discrepancy; Mekelle's study included 261 nurses, whereas the current study included 387 nurses. Likewise, the tool used in Mekelle's study is quite different, it was pain management for only children. However, this study employed a comprehensive tool regardless of age category. In a study done in Turkey, the sample size was 246 and the setting was training and research hospitals, whereas in our study the sample size was 387 and the setting was referral hospitals. In a study done in Australia, the sample size was 300 nurses, and the settings were general hospital and nursing home and knowledge of nurses towards pain management among the elderly, whereas in the current study the sample size was 387, and the setting was single-center referral hospital and knowledge of nurses towards pain management among all patients.

### 4.2. Attitude of Study Participants towards Pain Management

Half (51.7%) (95% CI (46.3%, 56.1%)) of the study participants had a favorable attitude towards pain management. This finding was consistent with studies done in Western Canada, 49% [26], in Zimbabwe, 56% [16], and in Jimma, Western Ethiopia, 49.8% [14]. 

This finding was lower than studies done in Malaysia, 78.5% [4], and in Uganda, 75% [18] in which study participants were having a favorable attitude towards pain management. The possible justification for this difference might be due to differences in study setting, the difference in sample size, different instruments used, and the difference in the outcome rating they used; for example, in Malaysian study, the knowledge was rated as high, moderate, and low. These may change the figurative interpretation of this study as good and poor knowledge. For training on pain management (75% of them trained in Malaysian study and 89% in Uganda), unlike the above study (Uganda), this study did not involve Doctorate nurses.

On the other hand, this finding was higher than the studies done in the United States of America (USA) [9] and in Hong Kong, China, where 27.7% and 44% [27] of registered nurses, respectively, had favorable attitudes towards pain management. The reason behind these differences might be study setting difference and the difference in tool/instruments used (the Nurses' Knowledge and Attitudes Survey Regarding Pain (NKASRP) was used to measure nurses' knowledge and attitudes regarding pain in the above Chinese study). Another justification may be the fact that, unlike in this study, in Hong Kong (China), only 1% of study participants had MSc and above in comparison to 3.6% in our study.

The study only assessed the descriptive quantitative part of pain management but not the qualitative and associated factors that independently affect nurses' knowledge and attitude towards pain management, which limits the current study.

## 5. Conclusion

This study revealed that nurses working at University of Gondar hospital had good knowledge (66.9%) and a lower level of favorable attitude (51.7%) towards pain management.

## 6. Recommendation

Based on the finding of this study, the researchers recommended the policy designers to educate and train nurses in pain assessment and management and continuous professional development to maintain or even improve knowledge and attitude towards pain assessment among nurses. It would be so important for prospective scholars to involve analytical study design to identify factors associated with the knowledge and attitude on pain management. Thus, the situation demands various educational and quality improvement initiatives that could enhance the nurse‘s knowledge and attitude in the area of pain management.

## Figures and Tables

**Table 1:** Sociodemographic characteristics of the study participants, University of Gondar comprehensive specialized referral hospital, Northwest Ethiopia, 2019 (*n* = 387).

Variables	Categories	Frequency	Percent (%)
Sex	Male	225	58.1
	Female	162	41.9

Age	19–29 years	308	79.5
	30–41 years	79	20.5

Marital status	Single	243	62.8
	Divorced	13	3.4
	Widowed	4	1.0
	Married	127	32.8

Years of experience	0–3 years	232	59.9
	4–7 years	140	36.2
	8–12 years	15	3.9

Qualification	Diploma	48	12.4
	BSc nurse	324	83.7
	MSc and above	15	3.9

Unit/ward	Emergency departments	90	23.3
	Outpatient departments	75	19.4
	Surgical ward	82	21.2
	Medical ward	69	17.8
	Intensive care units	5	1.3
	Others^*∗*^	66	17.1

^*∗*^Others = psychiatry ward, gynecology ward, and ophthalmic ward.

**Table 2:** Description of knowledge items among nurses about pain management, University of Gondar comprehensive specialized hospital, Northwest Ethiopia, 2019 (*n* = 387).

Questions	Categories	Frequency	Percentage (%)
Staff can always pick up cues from children that indicate that they are in pain	Yes^*∗*^	243	62.8
	No	144	37.2
Because narcotics can cause respiratory depression, they should not be used in pediatric patients	Yes^*∗*^	132	34.1
	No	255	65.9
It may often be useful to give a placebo to a patient in pain to assess if he is genuinely in pain	Yes^*∗*^	269	69.5
	No	118	30.5
Estimation of pain by an M.D. or R.N. is as a measure of pain as a patient's self-report	Yes^*∗*^	228	58.9
	No	159	41.1
Patients having severe chronic pain often need higher dosages of pain meds than patients with acute pain	Yes^*∗*^	160	41.3
	No	227	58.7
Distraction, for example, by the use of music or relaxation, can decrease the perception of pain	Yes^*∗*^	83	21.4
	No	304	78.6
Increasing analgesic requirements are signs that the patient is becoming addicted to the narcotic	Yes^*∗*^	102	26.4
	No	285	73.6
If a patient and/or patient family member reports that a narcotic is causing euphoria, she/he should be given a lower dose of the analgesic	Yes^*∗*^	131	33.9
	No	256	66.1
One fourth of patients receiving narcotics around the clock become addicted	Yes^*∗*^	127	32.8
	No	260	67.2
The preferred route of administration of narcotic pain relievers to patients with pain is IM	Yes^*∗*^	160	41.3
	No	227	58.7
Patients can be maintained in a pain free state	Yes^*∗*^	104	26.9
	No	283	73.1
Patients with chronic pain should receive pain medications at regular intervals with or without the presence of discomfort	Yes^*∗*^	141	36.4
	No	246	63.6

^*∗*^ = correct answer; IM: intramuscular.

**Table 3:** Description of attitude items among nurses towards pain management, University of Gondar comprehensive specialized referral hospital, Northwest Ethiopia, 2019 (*n* = 387).

Questions	Categories	Frequency	Percent (%)
Do you think that lack of pain expression does not mean lack of pain?	Strongly disagree	26	6.7
	Disagree	67	17.3
	Neutral	38	9.8
	Agree	144	37.2
	Strongly agree	112	28.9

Do you believe giving narcotics on a regular schedule is preferred over PRN schedule for continues pain?	Strongly disagree	38	9.8
	Disagree	59	15.2
	Neutral	52	13.4
	Agree	144	37.2
	Strongly agree	94	24.3

When a patient requests increasing amounts of analgesics to control pain, do you think that this usually indicates that the patient is psychologically dependent?	Strongly disagree	12	3.1
	Disagree	37	9.6
	Neutral	45	11.6
	Agree	164	42.4
	Strongly agree	129	33.3

Do you think a patient should experience discomfort prior to giving the next dose of pain medication?	Strongly disagree	11	2.9
	Disagree	40	10.3
	Neutral	63	16.3
	Agree	156	40.3
	Strongly agree	117	30.2

Would you believe that patient receiving narcotics on a PRN basis may likely to develop clock-watching behaviors?	Strongly disagree	14	3.7
	Disagree	45	11.6
	Neutral	73	18.9
	Agree	159	41.1
	Strongly agree	96	24.8

Do you think that the most accurate judge of the intensity of the patient's pain is the patient him/herself?	Strongly disagree	9	2.4
	Disagree	25	6.5
	Neutral	64	16.5
	Agree	152	39.3
	Strongly agree	137	35.4

When a patient in pain is receiving analgesic medication on a PRN basis, should it be appropriate for the patient to request pain medications before the pain returns?	Strongly disagree	20	5.2
	Disagree	40	10.3
	Neutral	63	16.3
	Agree	165	42.6
	Strongly agree	99	25.6

Would you believe that as narcotics can cause respiratory depression, they should not be used in pediatric patients?	Strongly disagree	26	6.8
	Disagree	41	10.6
	Neutral	82	21.2
	Agree	137	35.4
	Strongly agree	101	26.1

Do you agree that children cry all the time; therefore, diversional activities are indicated rather than actual pain?	Strongly disagree	15	3.9
	Disagree	40	10.3
	Neutral	70	18.1
	Agree	162	41.9
	Strongly agree	100	25.8

Do you agree for that the most suitable dose of morphine for a patient in pain is a dose that best controls the symptoms; there is no maximum dose (i.e. a level that must not be)?	Strongly disagree	13	3.4
	Disagree	44	11.4
	Neutral	65	16.8
	Agree	157	40.6
	Strongly agree	108	27.9

If the cause of patient's pain is not known, opioid analgesics should not be given during pain management	Strongly disagree	11	2.9
	Disagree	48	12.4
	Neutral	65	16.8
	Agree	166	42.9
	Strongly agree	97	25.1

For effective pain treatment of cancer pain do you think that it is necessary to continuously assess the pain and the efficacy of therapy?	Strongly disagree	8	2.1
	Disagree	26	6.7
	Neutral	45	11.6
	Agree	175	45.2
	Strongly agree	133	34.4

Do you agree that it is the patient's right to expect total pain relief as a consequence of treatment?	Strongly disagree	15	3.9
	Disagree	23	5.9
	Neutral	43	11.1
	Agree	183	47.3
	Strongly agree	123	31.8

## Data Availability

The data used for this study are avilable in the document. Nevertheless, it is possible to access by contacting the corresponding author.

## Abbreviations

ANOVA: Analysis of variance
CI: Confidence interval
Epi Info: Epidemiological information
ICU: Intensive care unit
IM: Intramuscular
MD: Medical doctor
SD: Standard deviation
SPSS: Statistical package for the social sciences.
